# A conjugate of octamer-binding transcription factor 4 and toll-like receptor 7 agonist prevents the growth and metastasis of testis embryonic carcinoma

**DOI:** 10.1186/s12967-015-0524-y

**Published:** 2015-05-20

**Authors:** Guimiao Lin, Xiaomei Wang, Wanxian Yi, Chuanxia Zhang, Gaixia Xu, Xiaomei Zhu, Zhiming Cai, Yu Liu, Yuwen Diao, Marie Chia-Mi Lin, Guangyi Jin

**Affiliations:** The Institute of Urinary and Reproductive, the Engineering Lab of Synthetic Biology, School of Medicine, Shenzhen University, Shenzhen, 518060 China; Shenzhen Key Laboratory of Translational Medicine of Tumor, Cancer Research Center, School of Medicine, Shenzhen University, Shenzhen, 518060 China; College of Optoelectronic Engineering, Key Laboratory of Optoelectronics Devices and Systems of Ministry of Education/Guangdong Province, Shenzhen University, Shenzhen, 518060 P. R. China

**Keywords:** Immunotherapy, Embryonic carcinoma, OCT4, TLR7 agonist

## Abstract

**Background:**

The immune non-recognition is often the underlying cause of failure in tumor immunotherapeutic. This is because most tumor-related antigens are poorly immunogenic, and fail to arouse an efficient immune response against cancers. Here we synthesized a novel TLR7 agonist, and developed a safe and effective immunotherapeutic vaccine by conjugating this TLR7 agonist with the pluripotency antigen OCT4.

**Methods:**

Purified recombinant OCT4 protein was covalently linked with a novel TLR7 agonist to form a TLR7-OCT4 conjugate (T7-OCT4). After conjugation, the *in vitro* release of IL-12 and IFN-γ was observed in spleen lymphocytes. Mice were immunized with TLR7-OCT4, and the release of IFN-γ, the percentages of CD3+/CD8+ T cells and the OCT4-specific cytotoxicity rates were measured. The immunized mice were challenged with mouse embryonic carcinoma (EC), and the tumor volume and tumor weight were determined. Blood routine examination was performed to evaluate the biosafety of TLR7 agonist and TLR7-OCT4 conjugate in mice*.*

**Results:**

T7-OCT4 conjugate significantly increased the *in vitro* release of IL-12 and IFN-γ by mouse spleen lymphocytes. In addition, the release of IFN-γ, the percentages of CD3+/CD8+ T cells and the tumor-specific cytotoxicity rates in immunized mice were significantly higher. Importantly, in EC xenografted mice, immunization with T7-OCT4 conjugate decreased the growth of the tumor dramatically up to 90 %, as compared to mice immunized with OCT4 protein or TLR7 agonist alone. Furthermore, blood routine examination demonstrated that no abnormalities of the blood cells and components in the blood fluids were detected by T7-OCT4 and TLR7 agonist injections.

**Conclusions:**

Our results showed that conjugating OCT4 protein to the novel TLR7 agonist produced a vaccine which is effective and safe in preventing tumor growth in mice. Our results suggest that this type of vaccine formulation has great potentiality in preventive vaccines against OCT4 expressing tumors.

**Electronic supplementary material:**

The online version of this article (doi:10.1186/s12967-015-0524-y) contains supplementary material, which is available to authorized users.

## Background

OCT4 (Octamer-binding transcription factor 4), also known as POU5F1, is a member of the mammalian POU family of transcriptional factors. It functions as a key regulator of the self-renewal and pluripotency of embryonic stem (ES) cells [1]. OCT4 has been reported to be highly expressed in many tumors, such as carcinomas of breast [2], testis, bladder [3], germ-cell tumors, and in cancer stem cells [4]. Tumors with intense OCT4 expression have been associated with further disease progression, greater degree of metastasis, and shorter cancer related survival [5, 6]. Kavita *et al.* have shown that OCT4-specific T cells could be readily detected in freshly isolated T cells from over 80 % of healthy donors, as compared to 35 % of patients with newly diagnosed germ-cell tumors [7], suggesting that diminishing immune response to OCT4 may lead to the generation and development of germ-cell tumors. OCT4 is highly expressed in tumor cells, but is either absent or at very low level in a variety of normal cells [8], indicating that it is a potential tumor stem cell biomarker and an ideal target in cancer therapy [9].

Testis embryonic carcinoma (EC), a highly malignant germ-cell tumor, occurs primarily in young adults. EC cells, the malignant stem cells of teratoma, exhibit a striking over-expression of the stem-related protein OCT4 [4]. Thus, we hypothesized that OCT4 would be a very powerful inducer of immune response to EC tumors. As most protein, peptide and DNA antigens are poorly immunogenic and often fail to arouse an efficient immune response against cancers, it is important to find a strategy to enhance the immunogenicity of OCT4. Many studies have demonstrated that conjugation of Toll-like receptors (TLRs) ligands and antigens offer great advantages over non-coupled antigens [10]. TLRs are a family of proteins expressed in many types of cells including immune cells (DCs, macrophages, B cells, T cells et al.) and other tissue cells. TLR-4, TLR-5, and the heterodimers TLR-1/TLR-2 and TLR-2/TLR-6 are located on the cell surface. Conversely, TLR-3, TLR-7, TLR-8, and TLR-9 are located within endosomal compartments of the cell. TLRs are activated by appropriated ligands and the Toll-like receptor (TLR) signaling pathways play critical roles in many host immune defenses. TLRs recognize specific pattern molecules found in many microbial pathogens called pathogen-associated molecular patterns (PAMPs) and initiate signaling cascades to arouse immune responses which result in the eradication of invading pathogens. Thus, agonists of TLRs have aroused broad interest for the immunotherapy of cancer, because most of tumor cells express high levels of tumor-specific antigens [11, 12].

To date, at least 10 members of Toll-like receptors (TLRs) have been identified in human with different TLRs having different respective ligands. Of all TLRs, only TLR7/8 recognizes GU-rich short single-stranded RNA (ssRNA) as well as small synthetic molecules such as nucleoside analogues and imidazoquinolines. This creates the opportunity of screening and modifying highly efficient TLR7 agonists for the enhancing of immune response. Furthermore, TLR7/8 agonists not only activate the antigen-presenting cells (APCs), but also promote the activation of T cells and natural killer (NK) cells [13, 14]. Activation of TLR-7 leads to the engagement of MyD88, MAL, IRAKs, and IKKα, which promote IRF activation and the production of Type 1 IFNs and pro-inflammatory cytokines [15, 16]. In addition, synthetic imidazoquinoline-like molecules such as resiquimod (R-848) and imiquimod (R-837) have also been shown to activate NF-κB through TLR7 [17, 18].

Here we showed that a conjugate of exogenous purified recombinant OCT4 protein covalently linked to a TLR7 agonist (T7-OCT4) could elicit a surprisingly strong anti-tumor effect on EC tumors by inducing tumor-specific immune response. The mechanisms by which this T7-OCT4 complex promotes immune responses were investigated.

## Materials and methods

### Bacterial strains, plasmids, cell lines and mice

*Escherichia coli* (*E.coli*) strain BL21 and PGEX-KG-mouse-OCT4 were maintained in our lab. *E. coli* was regularly grown in Luria Broth (LB) media at 37 °C with shaking at 200 rpm or on media (supplemented with 100 μg/ml ampicillin LB_Amp_) solidified with 1.5 % agar. LLC Lewis lung cancer cells or mouse embryonic carcinoma F9 cells were maintained in our lab and cultured in Dulbecco's Modified Eagle's Medium (DMEM, Hyclone), supplemented with 10 % fetal bovine serum (FBS, Hyclone), 100 μg/mL penicillin (Ameresco) and 100 μg/mL streptomycin (Ameresco). Cells were cultured at 37 °C in a humidified atmosphere with 5 % CO_2_. BALB/c mice at 6 weeks of age were purchased from the Medical Laboratory Animal Centre of Guangdong Province, China. The experiments were carried out in accordance with recommendations cited in the Guide for the Care and Use of SPF (Special Pathogens free) Animal Center of Shenzhen University, Guangdong Province, China.

### Synthesis of TLR7 agonist

**Fig. 1:**
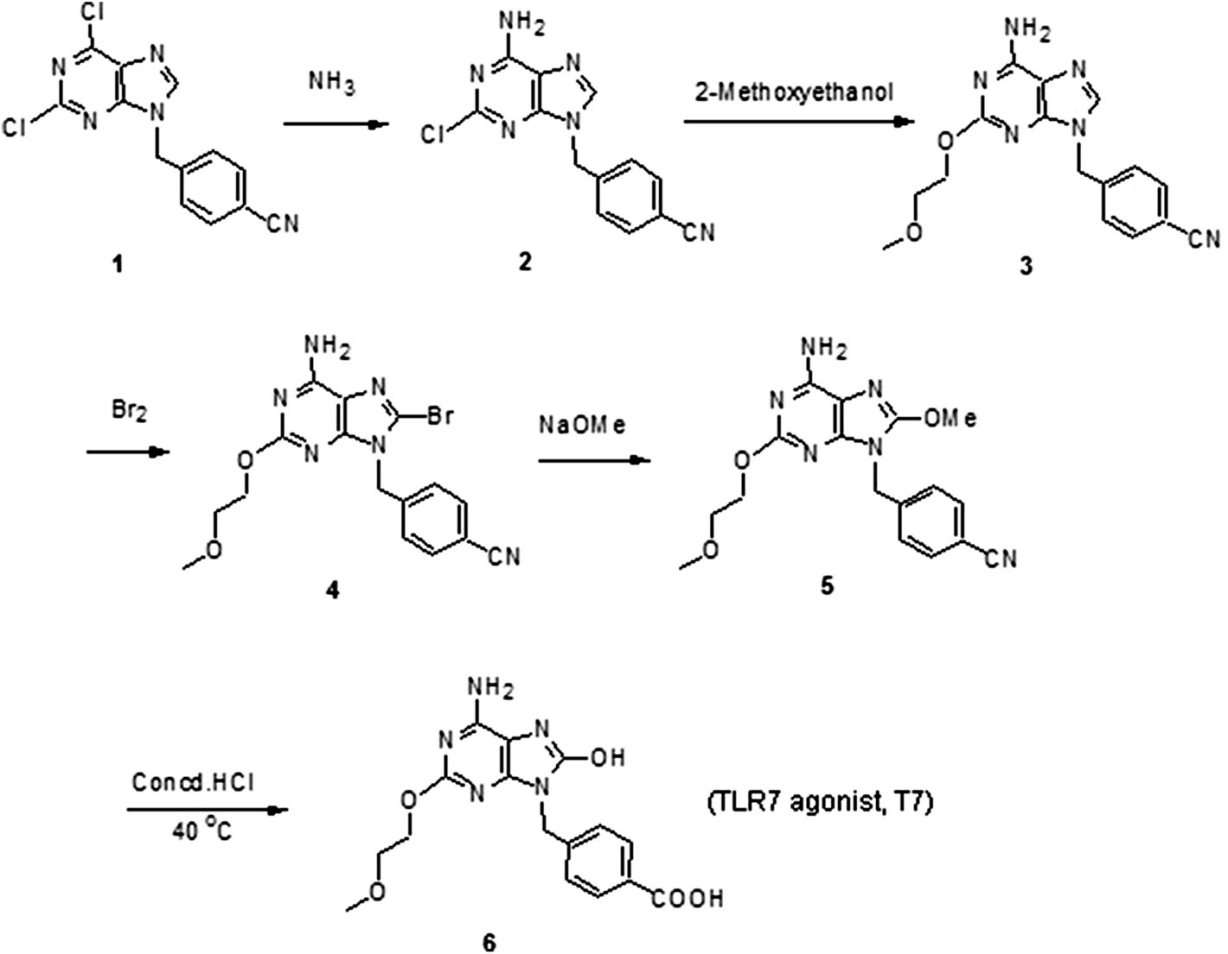
Schematic illustration on the synthesis of TLR7 agonist (T7)

The TLR7 agonist was synthesized in our laboratory, compound 5 was synthesized from 2,6-dichloropurine according to the published method [19, 20], The TLR7 agonist (compound 6) was separated from the hydrolysis of compound 5 in concentrated HCl (Fig. 1) as a free acid. Briefly, 100 mg of compound 5 was dissolved in 10 mL of concentrated HCl; the mixture was stirred at room temperature for 12 h, heated at 40 °C for 4 h; cooled to room temperature; and distilled under vacuum to remove the solvent. Then, the residue was dissolved in 10 mL of 5 % Na_2_CO_3_ in water, stirred for 30 min, and filtered. The clear filtrate was acidified with 5 % HCl at 5-10 °C, the precipitated product was filtered and washed three times with water, dried under vacuum at 60 °C, for 4 h to obtain the final product of TLR7 agonist (T7) as white powder. The structure of T7 was determined by Nuclear Magnetic Resonance assay (1H; 13C NMR).

### Production and purification of recombinant OCT4 immunogen and its conjugation with T7

Recombinant mouse OCT4 was produced by transfection of plasmid PGEX-KG-mouse-OCT4 into Bacterial strain BL21. The expressed protein was purified with glutathione sepharose beads (GE healthcare) at 4 °C, and assayed using a DC protein assay kit (BioRad). The benzenecarboxylic acid group on TLR7 agonist enabled facile coupling to protein with free amino using well established conjugation chemistry. To prepare a conjugate that could be administrated into mice and conveniently monitored spectrophotometrically, we chose EDC and NHS to activate carboxylic acid as active ester. The TLR7 agonist, EDC and NHS were mixed at 1:1:1 ratio in DMSO and incubated in the dark at room temperature overnight. Then the activated TLR7 agonist was added to OCT4 protein at 40-fold molar excess in PBS, and incubated at 4-8 °C overnight. To remove excess TLR7 agonist and catalyst EDC/NHS,the reaction mixture was washed with PBS three times on 10 000 MWCO Microcon filtration device (Millipore). The lyophilized conjugate was stable for at least 6 months at −20 °C.

### Stimulation of mouse spleen lymphocytes in vitro

Mouse spleen lymphocytes were isolated from BALB/c mice and grown in RPMI 1640 medium with 10 % FBS. Cells were seeded into 96-well plates at a density of 4 × 10^5^ cells per well. Different testing compounds were added to the cell culture medium at a final concentration ranging from 0.01 to 10 μM as indicated and incubated for 24 h. The levels of IL-12 and IFN-γ in cell culture supernatants were determined using enzyme-linked immunosorbent assay (ELISA) (eBioscience), according to the manufacturer’s instructions.

### Immunizations in mice

All experiments were routinely performed in groups of 8 mice each. On Day 1, 14, 28 and 42, mice were administrated (i.p.) PBS, OCT4 protein (15.6 μg in PBS per mouse), TLR7 agonist (1.9 μg in PBS per mouse), or T7-OCT4 conjugate (17.5 μg). Cells were harvested from spleens on the 3rd day after the last vaccination, and 2.5 × 10^5^ single cell suspensions from individual mice were incubated with 10 μg/ml OCT4 protein for 24 h. Then culture supernatants were collected and assayed for cytokine inductions by ELISA.

### Detection of CD8+ T lymphocytes

2.5 × 10^6^ spleen lymphocytes from immunized mice (PBS, TLR agonist, OCT4 and T7-OCT4) were seeded in 96-well plates and stimulated with 10 μg/mL OCT4 antigen or PBS alone every 24 h twice. Three days later, cells were collected and washed with PBST (0.05 %Tween in PBS), incubated with 5 % BSA for 1 h, stained with anti-CD4 FITC, anti-CD8 PE at 1 μg/ml final concentration on ice overnight, and then washed with PBST 3 times and immediately analyzed by flow-cytometry (Becton Dickinson).

### The CTL assays

Spleen from immunized mice were removed 3 days after the last injection, and single lymphocyte cell suspensions were obtained by teasing the organs through a sterile nylon mesh. Effector Cells (1.25 × 10^5^) were treated with 10 μg/mL OCT4 protein and again 24 h later. Cells were maintained for 3 days in a humidified 5 % CO_2_ incubator at 37 °C in RPMI 1640 medium containing 5 % fetal bovine serum in 96-well plates. Before the target cells (LLC or F9 cells) were added, 100 μl of supernatants in effector cells should have been removed.

Target cells were prepared by incubating LLC cells or F9 cells in tissue culture for 12–16 h. Target cells were diluted in RPMI 1640 with 5 % FBS and 10 mM HEPES at the concentration of 1x10^5^/ml and added 100 μl to effector cells in 96-well round-bottomed plates. After a 5 h incubation period at 37 °C, the levels of LDH in the supernatants released by target cells were determined according to the manufacturer’s instructions (Promega).

### Tumor prevention of T7-OCT4 vaccine in mice

All experiments were routinely performed in groups of 8 mice per group. Mice were immunized and challenged with tumors according to the method reported by Vani *et al.* [21]. Briefly, mice were administrated (i.p.) with PBS, OCT4 protein (15.6 μg in PBS per mouse), TLR7 agonist (1.9 μg in PBS per mouse), TLR7+ OCT4 (mixture of 15.6 μg OCT4 protein and 1.9 μg TLR7 agonist), or T7-OCT4 conjugate (17.5 μg) on Day1, 14, 28 and Day42. On Day35, mice were injected subcutaneously with 100 μl of F9 cell suspension (2 × 10^6^ cells) in the mid-back region, and sacrificed 14 days post-injection of F9 cells. Tumor xenografts were surgically dissected, weighed, and measured. Tumor volume was calculated according to the equation Length × Width^2^/2 with the length (mm) being the longer axis of the tumor. The generation of microvessels in tumors was observed using transparent dorsal skin fold window chamber (see Additional file 1). The experiments were carried out in accordance with recommendations cited in the Guide for the Care and Use of Laboratory Animals of Shenzhen University, Guangdong Province, China.

### Blood routine examination

Blood samples are harvested from mice immunized with PBS, OCT4 protein, TLR7 agonist, or T7-OCT4 conjugate on the 42th days. Blood was collected in anticoagulant tubes from the orbital sinus by removing the eyeball from the socket quickly. Blood routine examination was performed using a routine blood test instrument (Mindray RJ-0C107223) in the affiliated hospital of Shenzhen university by an experienced clinical doctor.

### Statistical analysis

All data were presented as mean ± SD. The results were compared by an analysis of variance. All statistical calculations were performed with Excel software package. A p-value < 0.05 was regarded as statistically significant difference.

## Results

### Synthesis of TLR7 agonist

TLR7 agonist (T7) was synthesized in our laboratory in six steps according to the scheme presented in Fig. 1. Mp: 280--282 °C; MS (ESI): m/z 360.30(MH^+^); and the 1H, 13C NMR were consistent with the structure assigned (see Additional file 2: Figure S1).

### Conjugation of TLR7 agonist to recombinant OCT4 protein

**Fig. 2:**
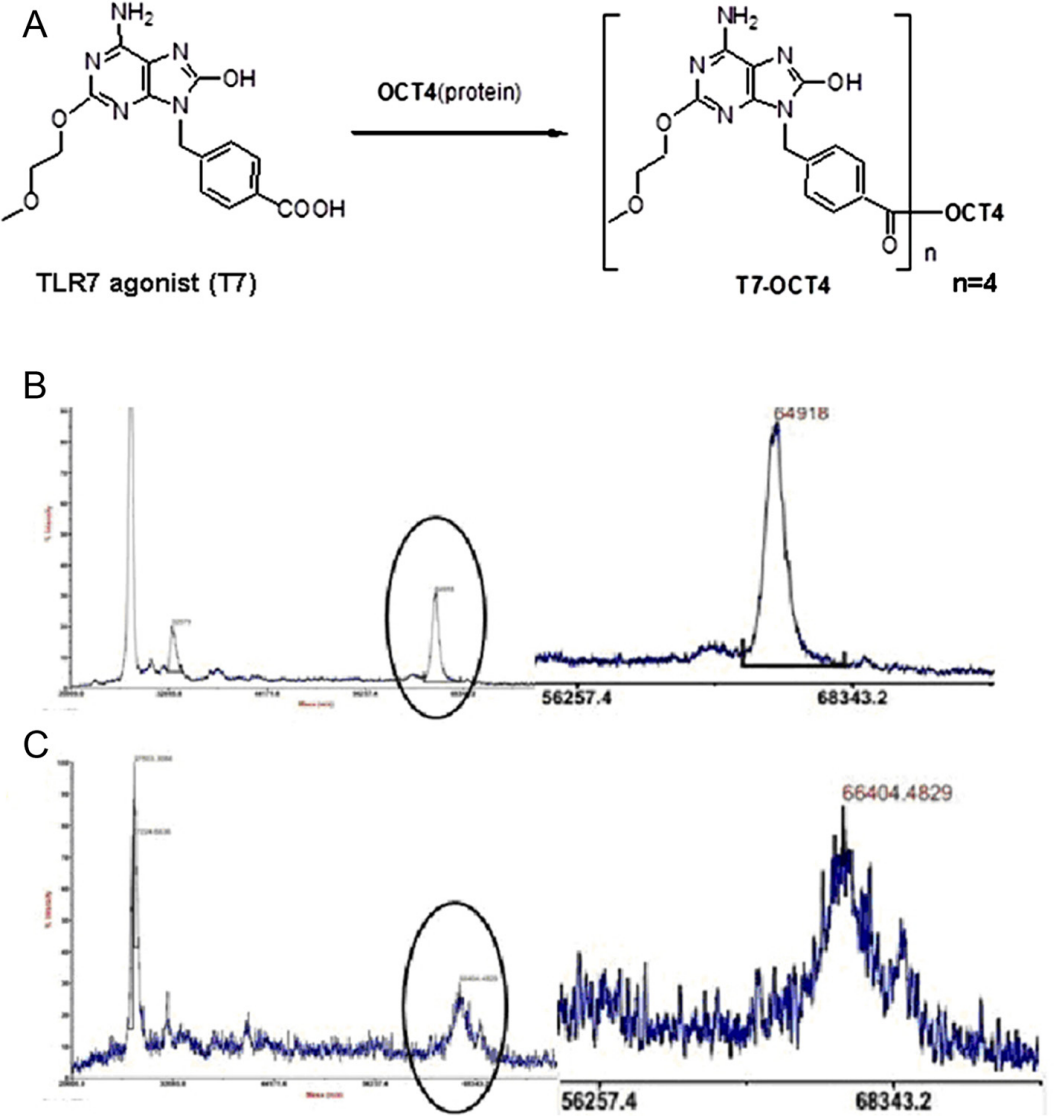
Quantification of TLR7 agonist conjugated to OCT4. **a**: Schematic illustration on the conjugation of TLR7 agonist to protein. **b**: The molecular weight of OCT4 before conjugation by mass spectrometry. **c**: The molecular weight of OCT4 after conjugation to T7 agonist by mass spectrometry. The molecular ratio of TLR7 agonist: OCT4 is 4:1

TLR7 agonist (T7) could be easily coupled to proteins and peptides antigens through the amide banding commonly used in peptide chemistry. In this study, we coupled T7 to OCT4 protein directly by NHSA-EDC procedure, yielded the conjugated antigen (T7-OCT4) (Fig. 2a) which was identified and characterized by MS spectrum (Fig. 2b). The ratio of T7 and OCT4 was determined to be 4:1. The molecular weight of TLR7 agonist was 359 Da, and the molecular weight of OCT4 protein before and after conjugation were 64.9 KDa and 66.4 KDa, respectively.

### T7- OCT4 induced potent cytokine release in vitro in spleen derived lymphocytes

**Fig. 3:**
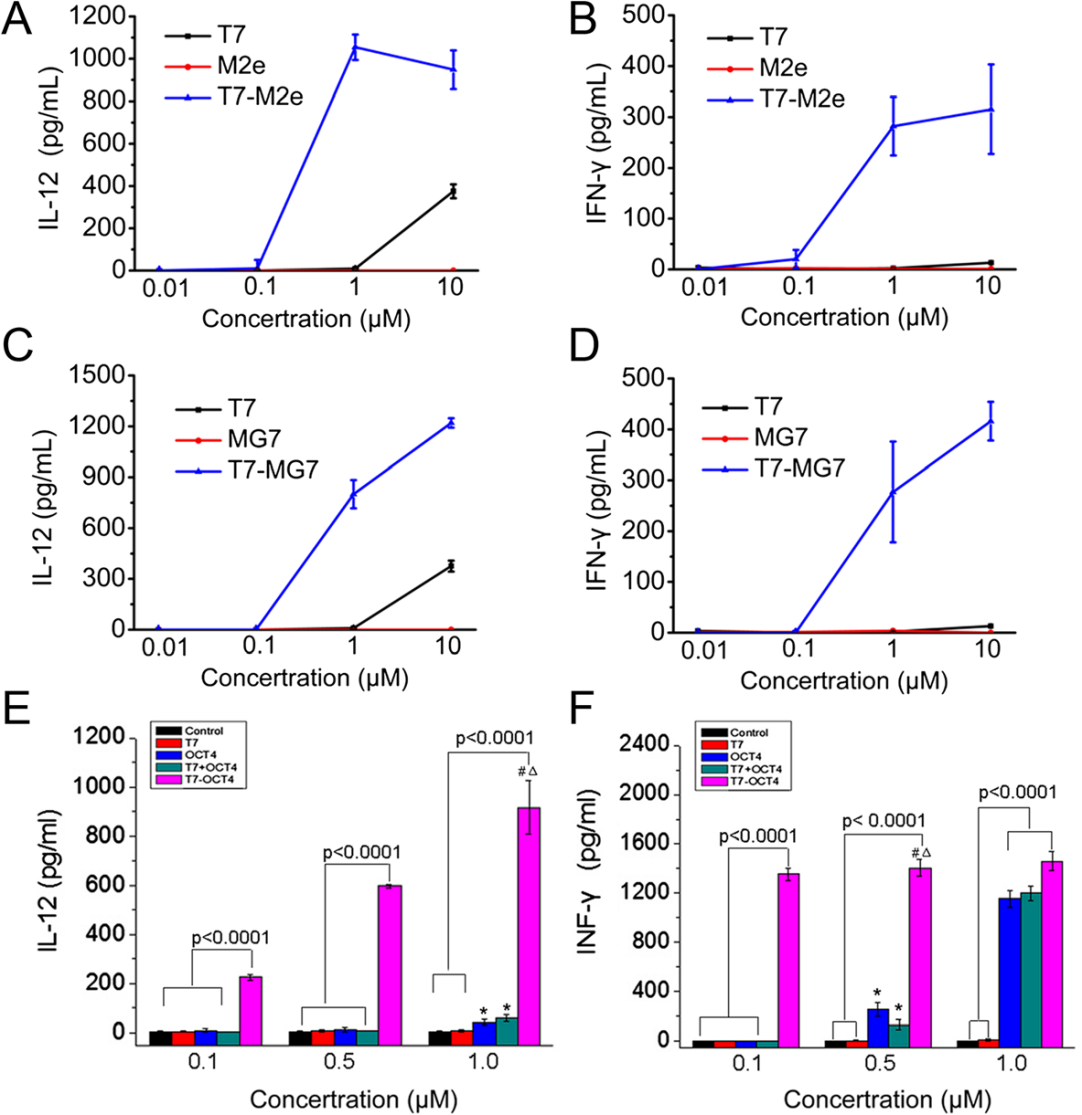
*In vitro* cytokine release in response to T7-OCT4 conjugates. Total murine spleen lymphocytes were isolated and treated with T7 agonist or T7 conjugates at various concentrations as indicated. Culture supernatants were harvested 24 h later, and cytokine levels were measured by immunoassay. The results are a representative of at least two separate experiments in triplicate per treatment. (**a** and **b**) Levels of IL-12 and IFN-Ƴ released by spleen lymphocytes treated with T7 agonist, M2e peptide and T7-M2e conjugate. (**c** and **d**) Levels of IL-12 and IFN-Ƴ released by spleen lymphocytes treated with T7 agonist, MG7 peptide and T7-MG7conjugate. **e** and **f**: Levels of IL-12 and IFN-Ƴ released by spleen lymphocytes treated with PBS, T7 agonist, OCT4 protein, T7 + OCT4 mixture and T7-OCT4 conjugate. Values are the means ± SD, n = 4; *, P < 0.01 vs Control;#, P < 0.01 vs OCT4; Δ,P < 0.01 vs T7 + OCT4. (Note: The concentration in the Figure describes the molarity of T7 agonist. The molarity ratio of OCT4 protein: T7 agonist = 1:4)

Since the TLR7 agonist we used is not commercially available, we first evaluate the immunological activity of this TLR7 agonist and determine the optimal dose. TLR7 agonist was conjugated to small peptides M2e or MG7 (T7-M2e or T7-MG7). M2e peptide (sequence MSLLTEVETPTRNEWECRCSDSSD) is the 24 amino-acid extra-cellular domain of M2 protein (M2) which is one of the most conserved antigens of influenza A virus. MG7 peptide (KPHVHTK) is a mimic epitope recognized by specific monoclonal antibody against gastric cancer associated antigen MG7. Both M2e and MG7 peptides are poor-immunogenic, as they are short peptides with low molecular weight. After incubation of spleen-derived lymphocytes with antigen, the release of cytokine IL-12 and IFN-γ were determined by ELISA. As shown in Fig. 3a-d, T7-M2e or T7-MG7, could stimulate substantially higher levels of cytokines release, as compared to TLR7 agonist alone (p < 0.001), indicating that the T7-M2e or T7-MG7 (0.1-1.0 μM) conjugates could activate the immune response more efficiently. T7 agonist alone could stimulate lymphocytes cytokine release at concentrations over 1 μM (p < 0.001), but not below 1 μM.

Next, we conjugated TLR7 agonist to the purified OCT4 protein to produce T7-OCT4 conjugate and measured its effect on the IL-12 and IFN-γ released by the lymphocytes. The total amount of T7 in unconjugated and conjugated is equal. The molarity of OCT4 is 1/4 of T7 agonist since the ratio of conjugation between TLR7 and OCT4 is 4:1. As shown in Fig. 3e and f, the T7-OCT4 induced significantly higher levels of IL-12 and IFN-γ release than that of control and T7 at indicated concentrations (0.1 μM, 0.5 μM, 1.0 μM) (p < 0.0001). Moreover, T7-OCT4 induced obviously higher levels of IL-12 than OCT4 and T7 + OCT4 at concentrations of 0.1 μM (p < 0.0001), 0.5 μM (p < 0.0001) and 1.0 μM (p < 0.01). Similarly, T7-OCT4 induced remarkably higher levels of IFN-γ than OCT4 and T7 + OCT4 at concentrations of 0.1 μM (p < 0.0001) and 0.5 μM (p < 0.01). OCT4 and T7 + OCT4 did not cause significant change in IL-12 at concentrations of 0.1 μM and 0.5 μM, and did not cause significant change in IFN- γ release at concentrations of 0.1 μM, however, OCT4 and T7 + OCT4 at 1.0 μM caused a significant increase of IL-12 (p < 0.01) and IFN- γ (p < 0.0001) release. This is possibly due to the non-specific immune response caused by high concentration of OCT4 antigen. Taken together, these results suggested that T7-OCT4 at the concentrations of 0.1-1.0 μM (the morality of T7 agonist) could effectively stimulate lymphocytes immune response.

### Tumor-prevention effect of T7-OCT4 conjugate in vivo in BALB/c mice xenografted with EC tumor

**Fig. 4:**
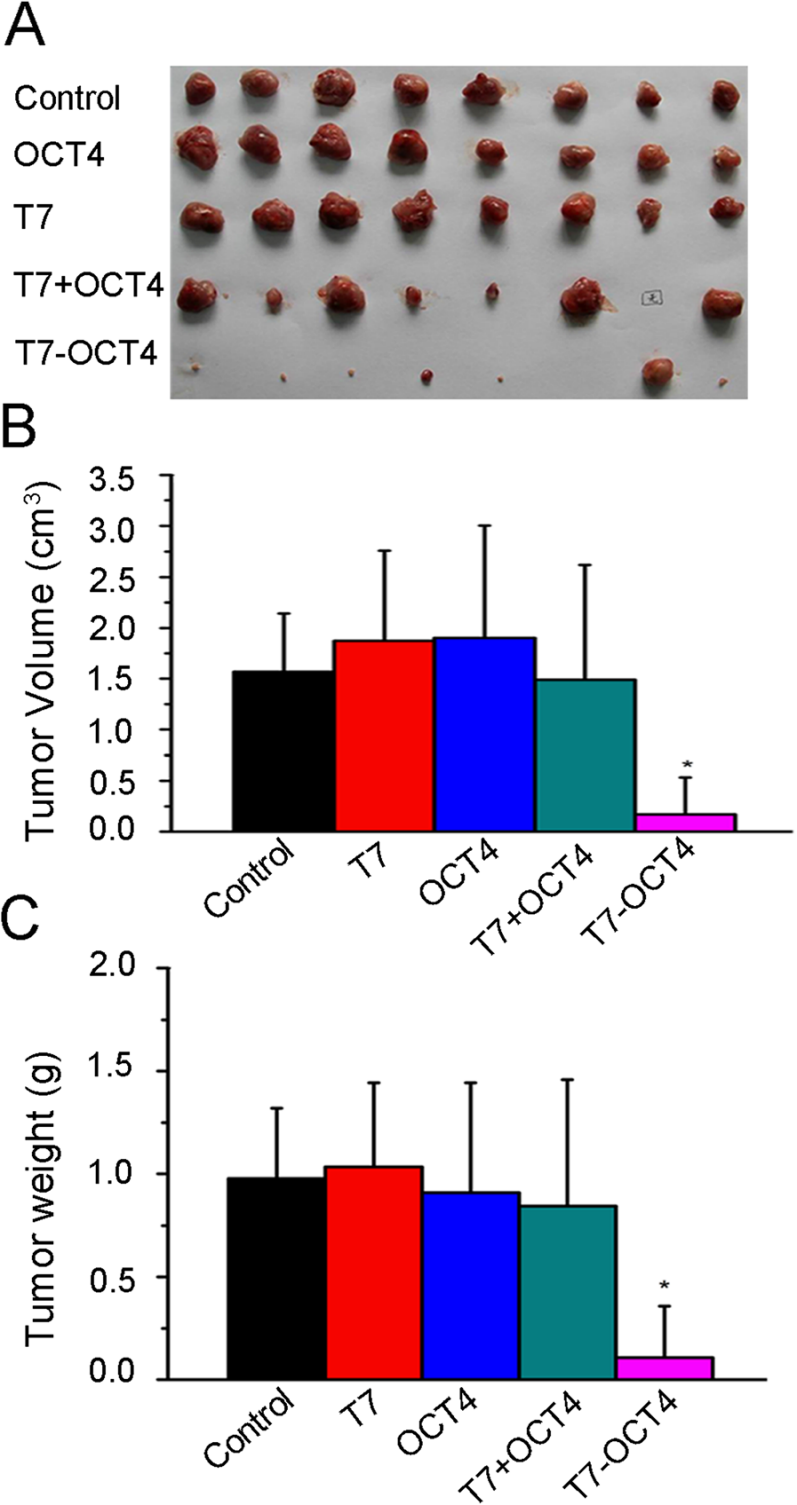
T7-OCT4 conjugates inhibits xenograft tumor growth **a**: Representative images of tumors derived from group Control, T7, OCT4 and T7-OCT4. 2x10^6^ F9 cells were injected subcutaneously into the mid-back area of adult BALB/c mice. **b**: Tumors were measured in three dimensions on day 14 post-injection of F9 cells. Values are the means ± SD, n = 8; *, P < 0.001 vs Control. **c**: Weight of xenograft tumors on day 14 post-injection of F9 cells. Values are the means ± SD, n = 8; *, P < 0.001 vs Control

To observe the *in vivo* tumor-prevention effect of T7-OCT4 vaccine, BALB/c mice were immunized with PBS, T7, OCT4, T7 + OCT4 (mixture of T7 and OCT4), or T7-OCT4 conjugate three times at biweekly intervals (On Day1, 14, 28). After 35 days, mice were injected subcutaneously with embryonic carcinoma F9 cells. On day 42, one more immunization was given. Fourteen days after post-injection of F9 cells, mice were sacrificed and tumor xenografts were surgically dissected, weighed, and measured. As shown in Fig. 4, immunization with T7, OCT4, as well as T7 + OCT4 did not produce significant growth inhibition as compared to that of control (*P > 0.05*). However, tumor growth was significantly inhibited by T7-OCT4 conjugate, the tumor volume and weight were dramatically decreased (*P < 0.001*). Using transparent dorsal skin fold window chamber, we found that tumors in T7-OCT4 conjugate treated mice showed decreased visible microvessels on Day 12 after tumor injection (as showed in Additional file 3: Figure S2), as compared to other treatment groups, which had abundant microvessels in the tumor mass as early as Day 7 after tumor-injection.

### T7-OCT4 vaccine induced tumor-specific immune responses in vivo in BALB/c mice

**Fig. 5:**
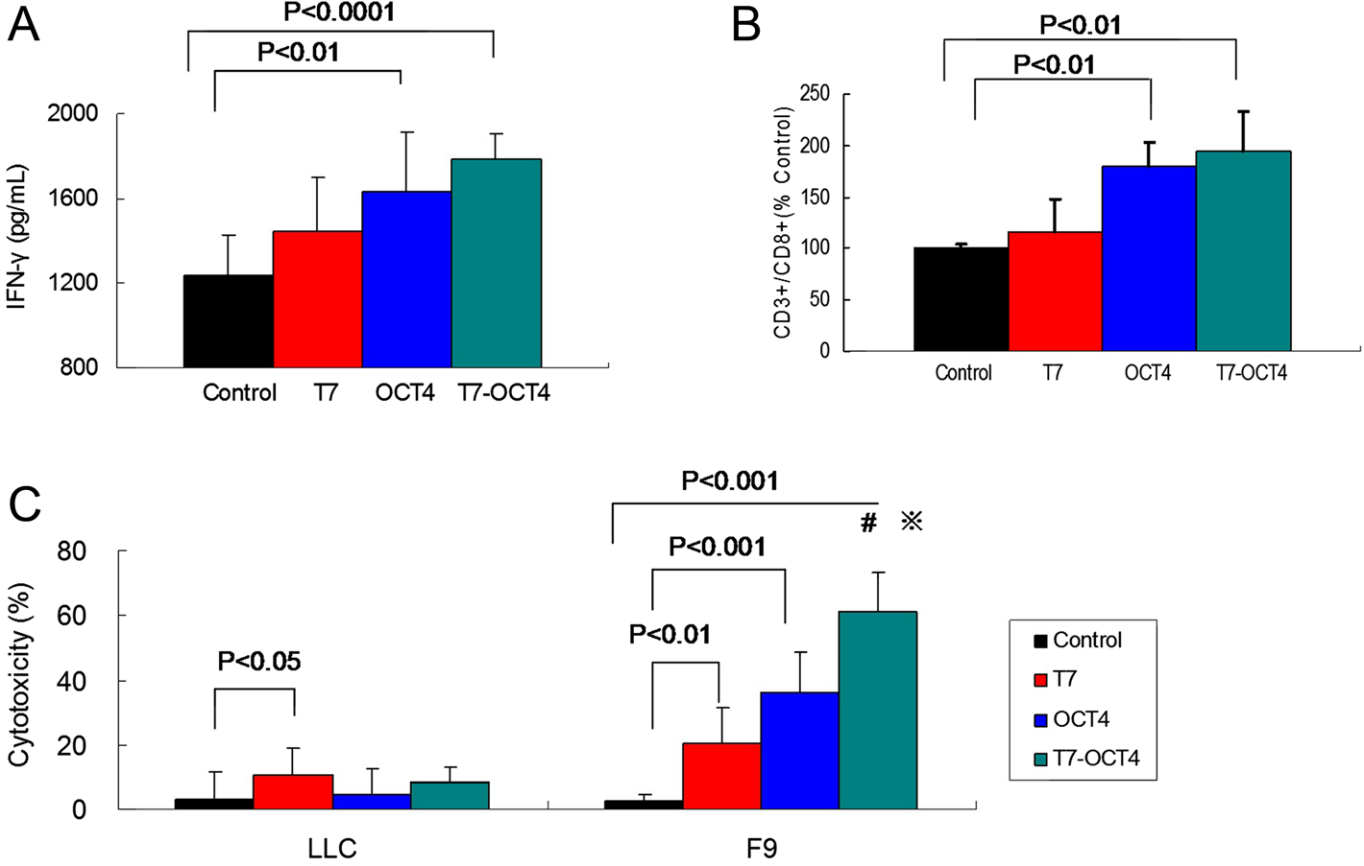
T7-OCT4 vaccine induced tumor-specific immune responses. Mice were i.p. immunized with different vaccines on Day 1, 14, 28 and 42. Three days after last immunization, **a**: Splenocytes were isolated and total splenocytes were re-stimulated with 10 μg/ml OCT4 antigen for 24 h, and the production of IFN-γ in culture supernatants was measured using ELISA. **b**: Total splenocytes were labelled with PE-anti-mouse CD8 and FITC-anti-mouse CD3, and the percentages of CD8+/CD3+ T cells in total splenocytes were measured using flow cytometry. **c**: Tumor-specific *in vitro* CTL response was measured as described in methods. *, P < 0.01 vs group T7; #, P < 0.01 vs group OCT4. Bars shown are mean ± SD (*n* = 5)

We immunized mice with T7-OCT4 on Day 1, 14, 28 and 42. Three days after the last immunization, lymphocytes were isolated from spleen, incubated with OCT4 antigen for 24 h, then the lymphocytes release of IFN-γ, the percentage of CD3+/CD8+ T cells, and the cytotoxicity to tumor cells were determined. As shown in Fig. 5a, the levels of IFN-γ in both T7-OCT4 and OCT4 groups were significantly higher than that of control and T7 groups. Consistently, the percentages of CD3+/CD8+ T cells are also remarkably higher (Fig. 5b). In addition, the lymphocytes induced cytotoxicity rates of F9 cells were highest in T7-OCT4 group, followed by T7 and OCT4 groups as compared to the control. As for the LLC Lewis lung cancer cells in which OCT4 antigen are absent, only T7 treatment showed increased cytotoxicity rates, while OCT4 and T7-OCT4 had no effect as compared to the control (Fig. 5c). These results indicated that T7-OCT4 vaccine can induced OCT4-specific immune responses, and the anti-tumor effect of T7-OCT4 are possibly centered on the activation of CD8 CTL that recognize specific OCT4 tumor antigen.

### Toxicity of T7 agonist and T7-OCT4 conjugate in mice

To evaluate the bio-safety of T7 agonist and T7-OCT4 conjugate *in vivo*, we performed the blood routine examination in animals administered with either PBS buffer, T7 agonist, OCT4 protein, or T7-OCT4 conjugate. The most important physiological system the administered compounds will interact is the blood fluids and their respective components (e.g. blood cells and hemoglobin). Therefore, it is crucial to determine the standard haematology markers. As shown in Table 1, blood test results showed no significant abnormalities in white blood cell count, (WBC), red blood cell count (RBC), haemoglobin (HGB), haematocrit (HCT), mean corpuscular volume (MCV), and platelet count (PLT). These results suggest that T7 agonist or T7-OCT4 did not cause any abnormalities of the blood cells and components in the blood fluids.

**Table 1:** Blood test results for treated BALB/c mice

Group	Control	T7	OCT4	T7-OCT4
WBC (10^9^/L)	7.735 ± 1.520	6.318 ± 0.685	6.391 ± 0.987	8.510 ± 2.081
RBC (10^12^/L)	11.02 ± 0.307	11.114 ± 0.169	11.15 ± 0.309	11.12 ± 0.210
HGB (g/L)	157.142 ± 5.460	157.428 ± 5.381	159 ± 5.099	158.428 ± 2.637
HCT (L/L)	0.486 ± 0.016	0.486 ± 0.013	0.484 ± 0.017	0.480 ± 0.007
MCV (10^−12^L)	44.142 ± 1.304	43.820 ± 1.494	43.383 ± 0.286	43.185 ± 1.076
PLT (10^9^/L)	481.333 ± 42.250	401.000 ± 84.052	452.285 ± 95.068	436.142 ± 51.181

Abbreviations: white blood cell count, (WBC), red blood cell count (RBC), haemoglobin (HGB), haematocrit (HCT), mean corpuscular volume (MCV) and platelet count (PLT)

## Discussion

Research underlying the immune non-recognition involved in the failure of tumor cells removal has led to the widespread exploration of immunotherapeutic approach aiming at the development of immune-mediated tumor destruction. To date, vaccines against pancreatic cancer (telomerase peptides) [22], lung cancer (MUC1) [23], breast cancer (HER2) [24], and prostate cancer (prostatic acid phosphatase, PAP) [25] have been tested clinically. In general, the clinical data clearly indicated that these vaccines are effective therapeutics. In this study, we used the pluripotency antigen OCT4 to activate the immune system against EC tumor cells and cancer stem cells, which are the root of tumor formation, recurrence and metastasis.

Although vaccine targeting OCT4 antigen is expected to achieve specific antitumor effect, especially on cancer stem cells and germ tumor cells. However, most tumor related antigens are poorly immunogenic, and often fail to arouse an efficient immune response against cancers. Consistently, our results also showed that although OCT4 protein alone can promote the generation of IFN-γ which are secreted by CD8^+^ T cells and CD4^+^ T cells(especially TH_1_ cells)*in vitro* and *in vivo*, however, immunization of OCT4 antigen alone *in vivo* could not efficiently inhibit the growth EC tumors, suggesting that although systemic immune response could be activated by OCT4 protein, no tumor-specific immune responses was observed *in vivo* during this process. It is possible that systemic immune activation by free OCT4 antigen does not create local cytokine and chemokine microenvironments required to mobilize immune cells to the site of tumors, or that the free OCT4 antigen induced immune cells could not effectively recognize the epitopes of OCT4 antigen expressed in EC tumor cells.

Several studies have demonstrated that conjugation of Toll-like receptors (TLRs) ligands and antigens produced better immunogenicity than non-coupled antigens [10, 26]. For example, Wille et al. reported that immunization with HIV-1 Gag protein conjugated to a TLR7/8 agonist resulted in a stronger generation of antigen-specific Th1 and CD8+ T cell responses than unconjugated HIV-1 Gag protein [27, 28]. Jason et al. found that immunization with the conjugate of whole OVA protein and TLR7 agonist resulted in around 1000-fold reduction in the frequency of bacteria in inoculated C57BL/6 mice, demonstrating a strikingly stronger ability to lower bacterial burden and control the infection [29].

Similarly, we designed and synthesized a novel TLR7 agonist (T7). This TLR7 agonist is small-molecule TLR7 ligands. It can functionalize many low immunogenicity antigens such as OCT4 to become potent immunostimulants by simple standardized conjugation method. The T7-OCT4 antigen serves as a model vaccine and could be used as a single immunotherapy, or in combination with other therapies for cancer prevention and treatment. Consistent to a previous study [26], our data showed that conjugation of TLR7 agonist and OCT4 protein could lead to rapid induction of inflammatory mediators IFN-γ and IL-12 in lymphocytes *in vitro*. The *in vivo* data showed that the T7-OCT4 vaccine can inhibit tumor growth by arousing tumor-specific immune responses. In summary, we synthesized a novel TLR7 agonist and conjugated with stemness antigen OCT4 to develop a safe and effective immunotherapeutic tumor vaccine. The vaccine is effectively against EC tumors and also expected to be useful for immunotherapy of tumor stem cells and other tumor cells which express OCT4 antigen. It is expected this vaccine will prevent the tumors from growth, spread and metastasis. Potentially, this vaccine may be of great value for the prevention of tumor generation after the transplantation of stem cells or induced pluripotent stem cells (iPS) into human body, as OCT4 is indispensible for most stem cells to generate tumors.

## Conclusions

Lack of immune recognition is the main cause of the tumor cells escaping immune surveillance and resisting eradication by the immune system. To address this issue, many cancer immunotherapy approaches are centered on the activation of CD8 CTL that recognizes specific tumor antigens. Here, we developed a novel TLR7 agonist and conjugated OCT4 protein covalently linked to this agonist (T7-OCT4). T7-OCT4 could elicit a surprisingly strong anti-tumor effect on embryonic carcinoma (EC) by inducing tumor-specific immune response in mice. Our results demonstrated that vaccination of OCT4 antigen conjugated to the novel TLR7 agonist is effective and safe against EC tumors and other tumors which express OCT4 antigen.

## Additional files

Additional file 1: **Transparent dorsal skin fold window chamber assay.**

Additional file 2: **Mass spectrometric analysis of TLR7 agonist.**

Additional file 3: **Optical observation of tumor microvascular generation.**
