# Supplementary material for: A conjugate of octamer-binding transcription factor 4 and toll-like receptor 7 agonist prevents the growth and metastasis of testis embryonic carcinoma
Source: J Transl Med. 2015 May 20;13:166. doi: 10.1186/s12967-015-0524-y (PMC4455914; doi:10.1186/s12967-015-0524-y)
Supplement: Supplementary file 1 — Transparent dorsal skin fold window chamber assay. [file 12967_2015_524_MOESM1_ESM.doc]

**Supplementary Information**

**Materials and Methods**

**1. Observation of tumor microvessels based on the dorsal skin fold window chamber**

BALB/c mice were bred and maintained in a SPF environment mentioned above. The surgical procedures were performed following the protocol described by Leunig *et al .* Briefly, prior to chamber implantation, the entire back of the animals was shaved and depilated after mice were anesthetized, and two symmetrical titanium frames were implanted so as to sandwich the extended double layer of skin. The body temperature of mice was kept around 37℃ and animals were allowed to recover for 24 h from anesthesia and microsurgery. For implantation of F9 cells, the coverslip of the chamber was removed and 2 µl of a dense tumor cell suspension from the cell culture were inoculated onto the upper tissue layer of the chamber and the chamber was closed again by the coverslip. Then the growth of microvessels and tumors were observed and imaged by a stereomicroscope (Olympus).

**Figure legends**

Figure S1: The 1H and 13C NMR spectra of compound 6 (TLR agonist, T7)

Figure S2: Optical observation of tumor microvascular generation based on the dorsal skin fold window chamber. The generation of microvessels was observed and imaged by a stereomicroscope at indicated days.

Reference:

1. Leunig M, Yuan F, Menger MD, Boucher Y, Goetz AE, Messmer K, et al. Angiogenesis, microvascular architecture, microhemodynamics, and interstitial fluid pressure during early growth of human adenocarcinoma LS174T in SCID mice. Cancer Res.1992;52:6553-60.
