# Supplementary figures and images for: A conjugate of octamer-binding transcription factor 4 and toll-like receptor 7 agonist prevents the growth and metastasis of testis embryonic carcinoma
Source: J Transl Med. 2015 May 20;13:166. doi: 10.1186/s12967-015-0524-y (PMC4455914; doi:10.1186/s12967-015-0524-y)

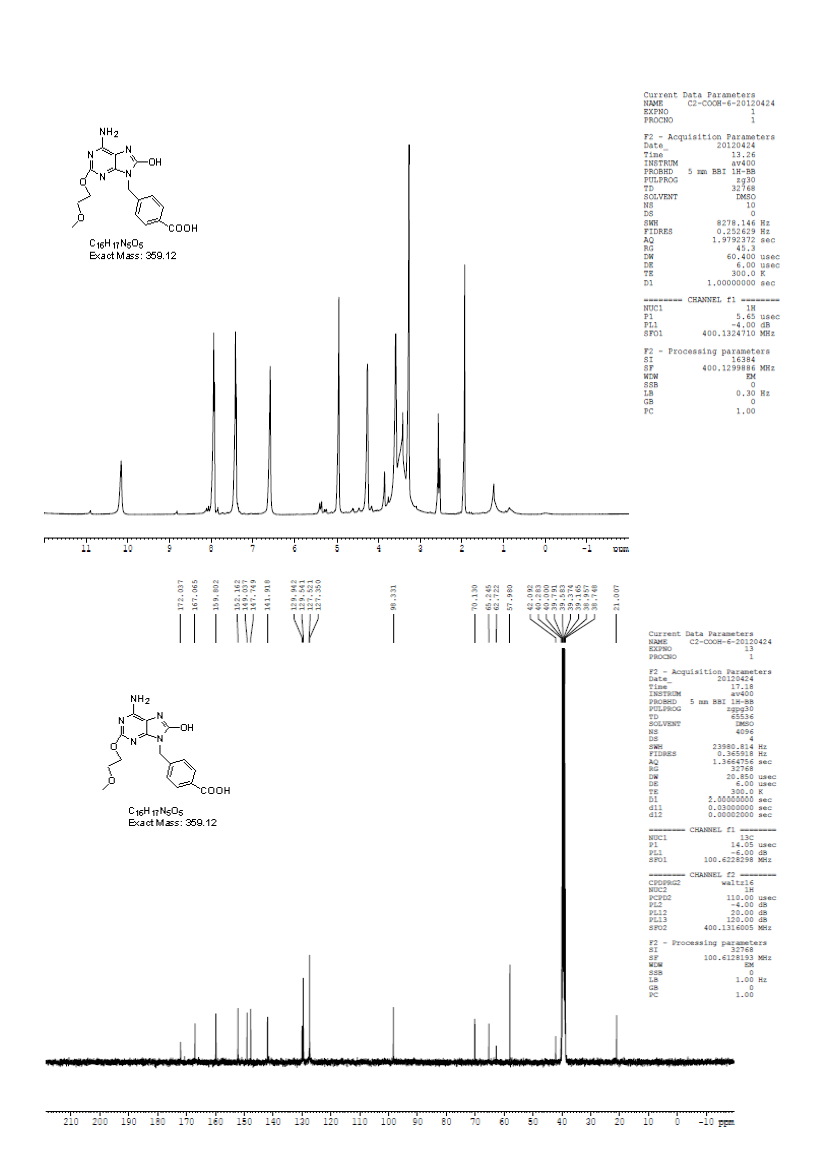

Supplement: Supplementary file 2 — Mass spectrometric analysis of TLR7 agonist. [file 12967_2015_524_MOESM2_ESM.tiff]

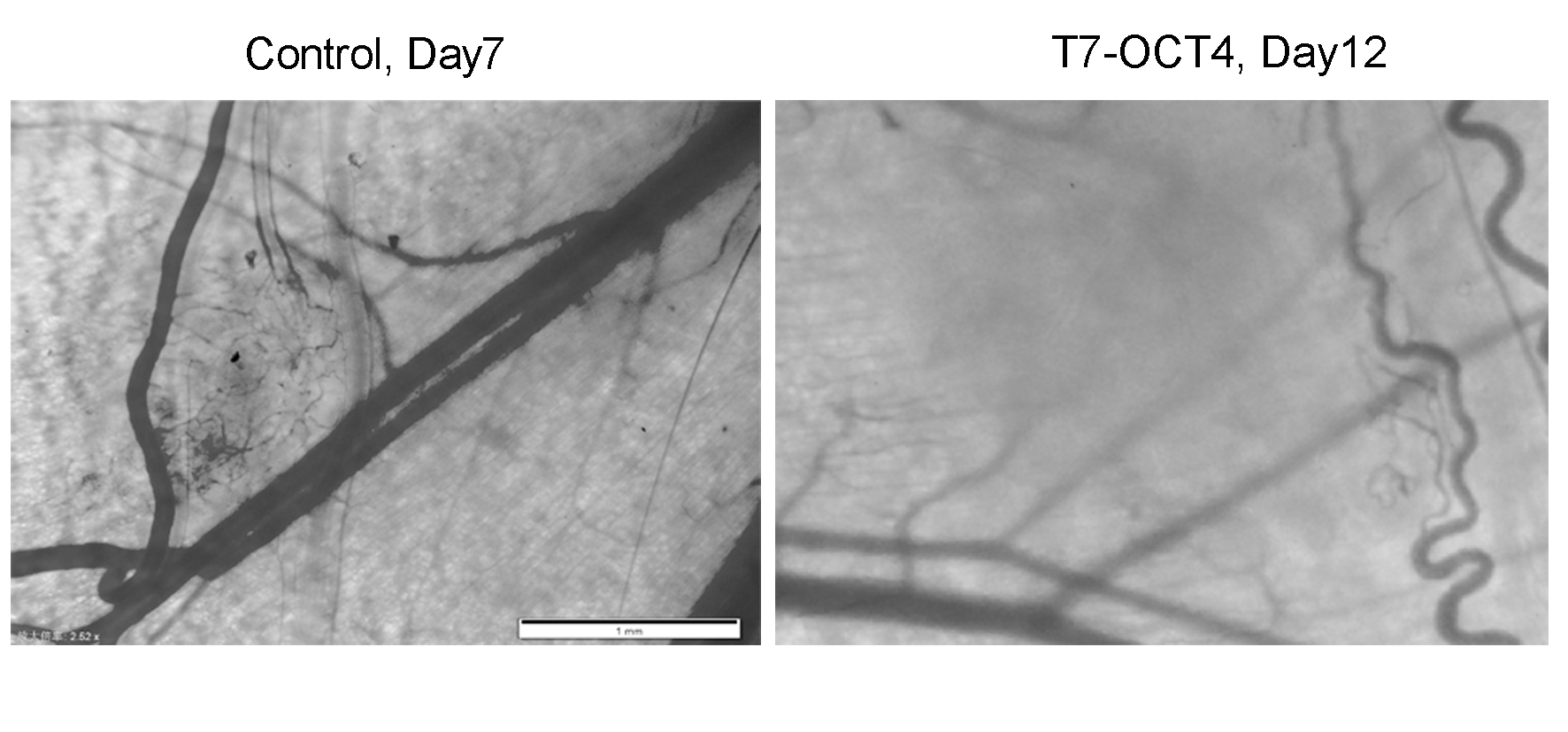

Supplement: Supplementary file 3 — Optical observation of tumor microvascular generation. [file 12967_2015_524_MOESM3_ESM.tiff]
